# Reliability and validity of a novel Kinect-based software program for measuring posture, balance and side-bending

**DOI:** 10.1186/s12891-017-1927-0

**Published:** 2018-01-08

**Authors:** Wilhelmus Johannes Andreas Grooten, Lisa Sandberg, John Ressman, Nicolas Diamantoglou, Elin Johansson, Eva Rasmussen-Barr

**Affiliations:** 1 Karolinska Institutet, Department of Neurobiology, Care Sciences and Society, Division of Physiotherapy, Huddinge, Sweden; 2 Functional Area Occupational Therapy & Physiotherapy, Allied Health Professionals Function, Karolinska University Hospital, 171 76 Stockholm, Sweden; 3 Sports medicine clinic, Bosön, Swedish Sports Confederation Centre, Lidingö, Sweden; 4 Ryggkirurgiskt Centrum, Stockholm, AB Sweden; 5 Faculty of Health and Occupational Studies, Department of Occupational and Public Health Sciences, Centre for Musculoskeletal Research, University of Gävle, Gävle, Sweden

**Keywords:** Balance, Kinect, Low back pain, Movement screening, Physical therapy, Psychometrics, Posture

## Abstract

**Background:**

Clinical examinations are subjective and often show a low validity and reliability. Objective and highly reliable quantitative assessments are available in laboratory settings using 3D motion analysis, but these systems are too expensive to use for simple clinical examinations. Qinematic™ is an interactive movement analyses system based on the Kinect camera and is an easy-to-use clinical measurement system for assessing posture, balance and side-bending. The aim of the study was to test the test-retest the reliability and construct validity of Qinematic™ in a healthy population, and to calculate the minimal clinical differences for the variables of interest. A further aim was to identify the discriminative validity of Qinematic™ in people with low-back pain (LBP).

**Methods:**

We performed a test-retest reliability study (*n* = 37) with around 1 week between the occasions, a construct validity study (*n* = 30) in which Qinematic™ was tested against a 3D motion capture system, and a discriminative validity study, in which a group of people with LBP (*n* = 20) was compared to healthy controls (*n* = 17). We tested a large range of psychometric properties of 18 variables in three sections: posture (head and pelvic position, weight distribution), balance (sway area and velocity in single- and double-leg stance), and side-bending.

**Results:**

The majority of the variables in the posture and balance sections, showed poor/fair reliability (ICC < 0.4) and poor/fair validity (Spearman <0.4), with significant differences between occasions, between Qinematic™ and the 3D–motion capture system. In the clinical study, Qinematic™ did not differ between people with LPB and healthy for these variables. For one variable, side-bending to the left, there was excellent reliability (ICC =0.898), excellent validity (*r* = 0.943), and Qinematic™ could differentiate between LPB and healthy individuals (*p* = 0.012).

**Conclusion:**

This paper shows that a novel software program (Qinematic™) based on the Kinect camera for measuring balance, posture and side-bending has poor psychometric properties, indicating that the variables on balance and posture should not be used for monitoring individual changes over time or in research. Future research on the dynamic tasks of Qinematic™ is warranted.

## Background

Visual observation is one of the most common tools used in the clinical examination of a patient and on a routine basis, clinicians observe and assess patients’ posture visually [1]. Visual observations are also used to examine the patients’ balance and active movements. Visual observations of balance and active movements are challenging, since these movements are fast and, in order to analyse posture, the therapist needs to observe the patient from different directions at the same time. Hence, these clinical examinations are highly subjective and often show a low validity and reliability [2–4]. Instead of these subjective analyses, objective and highly reliable quantitative assessments are obtainable in laboratory settings using 3D motion analysis and force plates [2]. However, 3D systems are too expensive for the observation of simple functional movements. Thus, there is a need to develop simpler yet more objective ways of assessing posture and balance in the clinic [2].

Microsoft released Kinect for Xbox 360 as a game controller in which a subject interacts with their console or computer by means of body movements. This low-cost sensor is able to track the contours of the human body in 3D, and has been used in motion capture analysis in clinical settings [5–7] together with a software program that enables the calculation the centre of mass (CoM) of the body. By projecting the CoM to the floor (base of support), the position, displacement, velocity and acceleration of the centre of pressure (CoP) can be calculated and is sometimes used as an estimation of postural control [8]. Using this camera together with a refined software program (Quickposture™), an interactive movement analysis system “Qinematic™” was developed to bridge the gap between subjective clinical examinations and objective advanced whole body motion analysis [9]. Qinematic™ seems to have the potential to play an important role in clinical assessment of movements and movement screening, and has been employed during recent years in different projects in sports and rehabilitation clinics in several countries in Europe, and in Australia. Before this novel measurement system can be implemented in modern physical therapy or other disciplines, the specific psychometric characteristics of the test situation should be established; i.e. the reliability and validity of Qinematic™ [10]. Reliability is defined as “the degree to which the measurement is free from measurement error” [11] and, in general, two factors affect test-retest reliability: 1) the variability of the person performing the movements and 2) the variation of the measuring device/observer. Validity is defined by the COSMIN panel as “the degree to which an instrument truly measures the construct(s) it purports to measure” [11]. There are different types of validity that should be distinguished; e.g. face validity, content validity, criterion validity, construct validity and discriminative validity. The purpose was to establish the test-retest reliability (absolute and relative) and construct validity of Qinematic™ for assessing balance, posture, and side-bending capacity in a healthy population. A further aim was to identify the discriminative validity of Qinematic™ in a setting of people with chronic LBP.

## Methods

### Design

This paper presents data from three different observational studies: (i) A test-retest reliability study of Qinematic™, in which the subjects performed one session of Qinematic™ on two occasions with approximately one week between the occasions; (ii) A construct validity study, based on three consecutive sessions of Qinematic™ simultaneously recorded with two systems: Qinematic™ and a motion caption system (BTS-Elite system) including one Kistler force plate. These two studies are clustered together and called the “laboratory-based studies”; (iii) A discriminative validity study, called “the clinical study”, in which Qinematic™ was used in a back clinic in which LBP patients were compared to healthy controls.

### Subjects

In the laboratory-based studies, we recruited 67 subjects (41 females, 26 males) in which 37 subjects were included in the reliability study (27 females, 10 males), and 30 subjects (14 females, 16 males) were included in the construct validity study. These were recruited through contacts and posters at Karolinska Institutet and Södertörns högskola in Flemingsberg, Stockholm. Subjects were included who were able to communicate in Swedish or English and were able to see, hear and understand the instructions from the computer screen. In the reliability study, the mean age was 34 years (SD = 12), mean weight and height was 70 kg (SD = 17) and 173 cm (SD = 7), respectively. In the construct validity study, the mean age was 42 years (SD = 14) and mean body weight and height were 75 kg (SD = 12.1) and 173 cm (SD = 7.2), respectively. A minority of the group had experienced pain during the previous week: 37% in the reliability and 27% in the construct validity study, while the majority of the group (58% and 81%, respectively) classified themselves as taking regular exercise, i.e. were physically active as defined as at least 30 min of physical activity/day five times per week. A written consent to agree to participate in the studies was obtained for all individual subjects. For those subjects below the age of 16, a written consent was obtained from their parents.

In the clinical study, a convenience sample of 20 patients (7 females, 14 males) with chronic LBP for at least 3 months was recruited from a physiotherapy clinic together with 17 healthy volunteers (9 females, 8 males). For both groups, the same inclusion criteria were used as in the laboratory-based study. To be included as a patient in this study, the symptoms should originate from the lower back without any radiations in the lower extremity. Controls were recruited through contacts among staff at an orthopaedic clinic. Only subjects reporting no disabilities or pain in the back, legs or feet during the previous three months before the trial were included. All subjects included had to be able to communicate in Swedish or English, and had to be able to see and understand the instructions on the computer screen. The mean age of the group was 40 years in the LBP group (SD = 9.2) and 41 years in the control group (SD = 6.9). The patients had a mean duration of pain of 2 years and rated their pain-related disability to 7.5 (mean) on the OSWESTRY scale.

### Qinematic™

The standard Qinematic™ movement screening test includes 7 different functional tasks and these were performed in the following order: #1. *Standing* with arms at the sides; instructions: “stand still”, #2. *Side-bending* to the left and right side with arms at the sides and palms facing the screen with forearms externally rotated; instructions: “Stand on both legs. Bend to the side as far as possible without your fingers touching your thigh”, #3. *Two-leg squat* with arms crossed over the chest; instructions: “bend your knees as if you are going to sit on a chair and get up again”, #4. *One-leg balance on right leg* with arms crossed over the chest; instructions: “lift your left leg in front of you, stand still for five seconds”, #5. *One-leg balance on the left leg* with arms crossed over the chest; instructions: “lift your right leg straight in front of you and stand still for five seconds”, #6. *One-leg squat, right* with arms crossed over the chest; instructions: “stand on your right leg and lift your left leg in front of you. Bend your right knee and rise up again”, #7. *One-leg squat, left* with arms crossed over the chest; instructions: “stand on your left leg and lift your right leg in front of you. Bend your left knee and rise up again”. These movements were grouped into four different sections: (A) Posture: movement #1, (B) Balance: Movements #1, 4 and 5; (C) Side-bending: #2, and (D) Movement Control: Movements #3, 6 and 7. Note: the psychometric properties for the section (D) movement control, will be published elsewhere, since the subjects included in the studies does not represent the population of interest (young healthy athletes with a risk for serious knee injuries; e.g. young female soccer players).

In Qinematic™, a subject is standing in front of the camera and follows oral and visual instructions to perform seven different functional movements. The subjects were standing in front of a computer screen (size 23 in.) about 3 m away from the screen, which was placed on a specially constructed cupboard in which also the Kinect camera was placed, around 82–86 cm from the floor. The subjects were informed of the test by instructions on a video clip and were subsequently told to perform the movement when ready by nodding their head. During the test, the information was given again, and they watched themselves on the screen as if in a mirror. If a movement was not conducted properly, due to a misunderstanding of the instructions, wrong positioning of the body parts or loss of balance, Qinematic™ detected this as a “no-go”, and the subject was asked to repeat this movement until it was performed acceptably. This procedure was repeated three times, and if Qinematic™ was still not satisfied with the performance, this movement was then recorded as a missing value. Subjects with missing values were omitted from the analyses.

### Procedures

Data for the laboratory-based studies was collected at the movement laboratory, division of physiotherapy, at the Karolinska Institutet in Stockholm during April/May 2016 for the construct validity study and during March/April 2017 for the reliability study. The subjects were dressed in shorts and singlet. The total time for each subject in the lab was 2 × 15 min for the reliability study and between 30 and 45 min for the construct validity study. The subjects filled in a questionnaire regarding demographics and background data and gave their informed consent before the test situation. In the reliability study, each subject performed the movements once, but on two different occasions with 6–8 days in between (md 7 days). The subjects were asked to retain their normal levels of physical activity and to report any specific event that occurred during the week that could have an influence on the second test. In the construct validity study, small spherical reflective markers (1 × 1 cm) were attached with sticky tape to various body parts. The markers on the lower extremity were divided into two halves, so as to minimize the interference with the Kinect camera. In the construct validity study, each subject performed the standard test three times (three trials) with a one-minute rest in between.

Data for the clinical study was collected at an orthopaedic clinic between February and April 2016. After filling in the background questionnaire and being given a clinical examination, the subjects were asked to change clothing into shorts and singlet and were then asked to perform the 7 Qinematic™ movements. The total time for the data collection was approximately 15 min/subject. The subjects performed the Qinematic™ test only once and received feed-back on the results directly after that.

### Apparatus

**Fig. 1:**
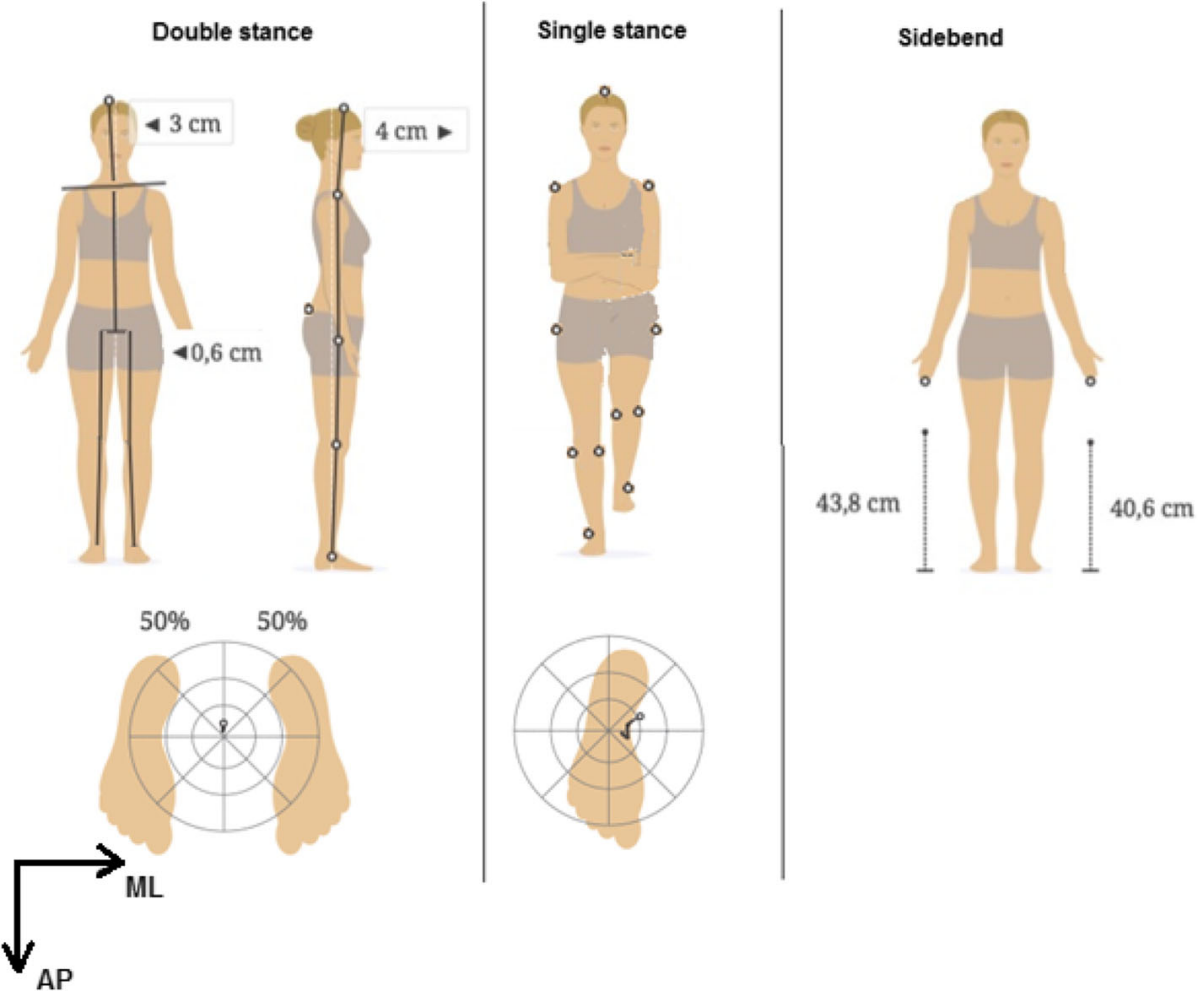
Movements and marker placement. Double stance, Single stance (right leg) and Side-bending

Qinematic™ (version 2.1.20) uses the Microsoft Kinect sensor (v2) to collect the data. This sensor samples data at a frequency of 30 Hz. In the laboratory studies, we used the BTS-Elite system which consists of an eight-camera motion analysis system (BTS-Elite 2002, version 2.8.4380; BTS, Milano, Italy) that records at 100 Hz the position of 15 spherical retro-reflective markers that were placed on the top of the head, the acromion (left & right), C7, the middle fingertips (left & right), the greater trochanters (left & right), mid-pelvic (the midpoint between Spina Iliaca anterior superior left and right), lateral and medial femoral epicondyles (left & right), the lateral malleolus (left & right) using double adhesive tape (Fig. 1).

Simultaneously with the kinematic data collection, ground reaction forces (GRF) were measured at 100 Hz using one Kistler force plate (0.50 m × 0.50 m) (Winterthur, Switzerland). Furthermore, two orthogonally placed digital video cameras recorded all the trials at 100 Hz in the sagittal and frontal plane from a distance of 2 m. These cameras were used in the synchronization process (see below). Three-dimensional trajectories of the markers were reconstructed manually using a tracking system (Tracklab-BTS-Elite, Milan, Italy). Kinematic and kinetic data were processed and filtered using a Butterworth low-pass filter with a cut-off at 7-Hz. A similar set-up was used in a previous study of our research group [12].

### Variables and data management

Qinematic™ provides 18 variables in their short report, and these were the variables of interest in the present study (Table 1). Six variables were related to posture (movement #1), nine variables related to balance (movements # 1, 4 and 5), and three variables related to side-bending (movement #2).

**Table 1:** Variables of interest used in the reliability (REL) and validity (VAL) studies

REL	VAL	Name	Abbreviation	Calculated in Qinematic™	Calculated in BTS-Elite	Unit
Posture						
R	C + D	Posture -Head-Lateral deviation	P-HL	Position of the top of the head in relation to the line of gravity in the frontal plane	Position of head marker in relation to midpoint between foot markers on Z-axis	Cm
R	C + D	Posture -Head-Frontal deviation	P-HF	Position of the top of the head in relation to the line of gravity in the sagittal plane	Position of head marker in relation to midpoint between shoulder (?) markers on X-axis	Cm
R	D	Posture - Neck angle forward	P-NA	The Cervicothoracic Angle forward, is the angle between the spine line and the neck line. The spine line is from the mid-hip to the mid-shoulder and the neck line is from the mid-shoulder to the top of the head. A positive number indicate that the neck is positioned forward (anterior) in relation to the spine and a negative number indicate that the neck is positioned backwards (posterior) in relation to the spine	Not calculated	Deg
R	C + D	Posture - Pelvic position	P-PP	Position of the mid-pelvic in relation to the line of gravity in the frontal plane	Position of midpoint of the markers on the trochanter marker in relation to midpoint between the foot markers on Z-axis	Cm
R	D	Posture - Height loss	P-HS	Estimated loss of body height minus measured body height:	Not calculated	Cm
R	C + D	Posture - Weight distribution	P-WB	Mean position of the Centre of mass in relation to the midpoint between ankle joints	Mean position of the CoP in relation to the midpoint between foot markers	%LEFT LEG
BALANCE- double stance (DS)						
R	C + D	Posture - Sway area	DS-SA	Sway area was calculated with Convex Hull-equation	Sway area was calculated with Convex Hull-equation	cm^2^
R	C + D	Posture - Maximal Sway Velocity in anterior-posterior (AP) direction	DS-SVAP	Velocity of the sway in AP-direction was calculated by ($v=\frac{\Delta s}{\Delta t}$)	Velocity of the sway in AP-direction was calculated by ($v=\frac{\Delta s}{\Delta t}$) and the 95:th percentile was used as a maximum value	cm/s
R	C + D	Posture - Maximal Sway Velocity in medio-lateral (ML) direction	DS-SVL	Velocity of the sway in ML-direction was calculated by ($v=\frac{\Delta s}{\Delta t}$)	Velocity of the sway in AP-direction was calculated by ($v=\frac{\Delta s}{\Delta t}$) and the 95:th percentile was used as a maximum value	cm/s
BALANCE- Single stance LEFT (SSL)						
R	C + D	Balance -Sway area	SSL-SA	Sway area was calculated with Convex Hull-equation	Sway area was calculated with Convex Hull-equation	cm^2^
R	C + D	Balance - Maximal Sway Velocity in anterior-posterior (AP) direction	SSL-SVAP	Velocity of the sway in AP-direction was calculated by ($v=\frac{\Delta s}{\Delta t}$)	Velocity of the sway in AP-direction was calculated by ($v=\frac{\Delta s}{\Delta t}$) and the 95:th percentile was used as a maximum value	cm/s
R	C + D	Balance - Maximal Sway Velocity in medio-lateral (ML) direction	SSL-SVML	Velocity of the sway in ML-direction was calculated by ($v=\frac{\Delta s}{\Delta t}$)	Velocity of the sway in AP-direction was calculated by ($v=\frac{\Delta s}{\Delta t}$) and the 95:th percentile was used as a maximum value	cm/s
Balance- Single stance RIGHT (SSR)						
R	C + D	Balance -Sway area	SSR-SA	Sway area was calculated with Convex Hull-equation	Sway area was calculated with Convex Hull-equation	cm^2^
R	C + D	Balance - Maximal Sway Velocity in anterior-posterior (AP) direction	SSR-SVAP	Velocity of the sway in AP-direction was calculated by ($v=\frac{\Delta s}{\Delta t}$)	Velocity of the sway in AP-direction was calculated by ($v=\frac{\Delta s}{\Delta t}$) and the 95:th percentile was used as a maximum value	cm/s
R	C + D	Balance - Maximal Sway Velocity in medio-lateral (ML) direction	SSR-SVML	Velocity of the sway in ML-direction was calculated by ($v=\frac{\Delta s}{\Delta t}$)	Velocity of the sway in AP-direction was calculated by ($v=\frac{\Delta s}{\Delta t}$) and the 95:th percentile was used as a maximum value	cm/s
Side-bending						
R	C + D	Fingertips Left - distance to floor/body height	SB-LF	Minimal distance of the left fingertip to floor divided by estimated height	Minimal distance to floor of marker on the left fingertip divided by self-reported height	%Body height
R	C + D	Fingertips Right - distance to floor/body height	SB-RF	Minimal distance of the right fingertip to floor divided by estimated height	Minimal distance to floor of marker on the right fingertip divided by self-reported height	%Body height
R		Weight distribution Left	SB-%BW	Maximal position of the CoM to the left in relation to the midpoint between ankle joints	Maximal position of the CoP to the left in relation to the midpoint between foot markers	%

*C* Construct validity *D* Discriminative validity. Abbreviations, ways of calculating the variables and units are presented

**Fig. 2:**
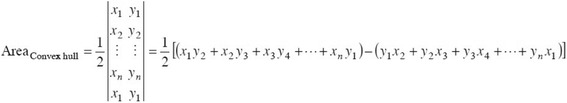
Convex Hull equation. x_1_ is the first value of COP displacement in A/P direction and y_1_ is the first value of COP displacement in M/L direction (x_2_, y_2_ is the second value, x_3_, y_3_ is the third value and so on)

In the reliability and clinical study, we used the data that was directly provided by the Qinematic™ software. In the construct validity study, Qinematic™, data was compared with the variables that were extracted from the BTS-Elite motion capture system aiming to mirror the Qinematic™ data. We used the Kistler force-plate to extract the position of the CoP and calculated from that the velocity (*v*) of the CoP in anterio-posterior (A/P) and medio-lateral (M/L) direction (*v* =∆s/∆t). We used the CoP trajectory to calculate the sway area during double- and single-leg standing in a similar way as Qinematic™ calculates sway area, i.e. using the Convex Hull equation (Fig. 2). However, in order to be able to compare the data from Qinematic™ with the data from the force plate during the single leg balance trials, we needed to synchronize the timing of the data collection. Qinematic™ starts measuring and analysing one-leg balance when the contralateral ankle (swinging leg) reaches its maximum in A/P direction and ends after 3–5 s (90–150 frames at 30 Hz) of balancing on one leg, which is shown in an animated video on the computer screen. To synchronize the force plate data with Qinematic™, kinematic data from the motion capture system (swinging leg in single-leg stance) together with the digital videos was used to define the displacement of the contralateral ankle (swinging leg) for each subject and by finding the maximum in A/P direction the start and stop of the movement could be defined. Note: for the variable max velocity, we used the 95th percentile instead of the absolute maximum value to eliminate possible outliers that could have occurred due to the mathematical calculations of the CoP.

### Statistics

#### Test-retest reliability

First, we tested whether there were any systematic differences between the two occasions with the Paired Wilcoxon Rank Sum test, using *p* < 0.05 as level of significance. Thereafter, we examined the relative reliability using the Intraclass Correlation Coefficient (ICC_[3.1]_),^1^ where we used a two-way mixed effects model [13]. We also calculated two absolute reliability statistics through the standard error of measurement (SEM)^2^ and the minimal detectable change (MDC),^3^ and these measures express the measurement error in the same unit as the original measurement for use on an individual level. The value of SEM reflects the uncertainty in the test score, while MDC represents the magnitude of change in an individual’s test score necessary to be sure that there is a real change in the construct and not a change due to measurement error. SEM and MDC are thus more clinically applicable measures than the ICC and are not affected by variability between subjects [14, 15]. The ICC-levels were classified as <20 = poor, 0.21–0.40 = fair, 0.41–0.60 = moderate, 0.61–0.80 = good, 0.81–1 = very good [16]. The SEM or MDC should preferably be as low as possible. Finally, we plotted Bland-Altman plots in order to detect any systematic difference or proportional bias [16].

Our hypothesis was that the posture, balance and side-bending performance could be repeatable, since video-based instructions were used (ICC > 0.6). On the other hand, the many different ways that a human being can perform the same functional movement suggests poorer reliability (ICC <0.4 and large SEM).

#### Construct validity

In the construct validity study, data from the third trial was used and, in case any data was missing, the second or first trial was used (in this order). The rationale for using three consecutive trials was to exclude a learning effect and to ensure that all subjects were performing the movements correctly. Each dataset was visually and statistically checked for normality by studying histograms and boxplots, comparing means and medians and by the Kolomogorov-Smirnov’s test and by the Shapiro-Wilk’s test. The data was not normal distributed, even after applying different logarithmic transformation and other transformations; hence non-parametric statistics were used. Another reason for not transforming the data is that the SEM and MDC should be expressed in their original units. The Paired Wilcoxon Rank Sum was used test to examine differences between the two datasets, using *p* < 0.05 as level of significance. We used scatterplots for visual examination for trends of any correlation between Qinematic™ and the motion capture system and if there were any outliers in the data. Spearman’s correlation tests were used to analyse strength of the correlation (22). Alpha level was set at *p* = 0.05 and the Spearman’s correlation coefficients were interpreted as follows: 0.0–0.3: negligible correlation, 0.3–0.5: low correlation, 0.5–0.7: moderate correlation, 0.7–0.9: high correlation, 0.9–1.0: very high correlation. Finally, Bland-Altman plots were also drawn to check for fixed and proportional bias (23).

Our hypothesis was that Qinematic was highly correlated (*r* > 0.7) with the motion capture system, as found in previous studies using the Kinect sensor [5, 6].

#### Discriminative validity

Discriminative validity concerns the ability of a measurement system to detect differences between two groups that are distinct differently from each other regarding the construct that is tested [11]. In the clinical study, the LBP group was compared with their healthy controls using Mann-Whitney U test using *p* < 0.05 as level of significance. Our hypothesis was that the variables measuring posture, balance and side-bending performance were poorer in the LBP group compared to the controls.

The analyses were carried out using Excel (Microsoft for Mac version 15.25.1) and SPSS Statistics (IBM version 23).

## Results

### Subjects

All subjects participated in the reliability and validity part of the laboratory-based studies (*n* = 37 and *n* = 30, respectively). In the clinical study, 20 LBP patients and 17 healthy controls were included. The subjects in the LBP group were significantly taller: 180 cm (SD = 8.6) compared to 173 cm (SD = 6.9) for the healthy controls, (*p* = 0.012). Another difference between the groups was that only 25% were classified as physically active in the LBP group compared to 59% in the healthy control group, but there were no differences between the groups regarding body weight; 82 kg in the LBP group compared to 75 kg in the healthy control group (*p* = 0.297).

In the laboratory-based studies, there was missing data for one subject concerning the measurements of the CoP during the double- and single leg balance trials and another subject’s data was missing concerning the side-bending trials in the reliability study. One subject used the wrong leg during a single leg stance task in the construct validity study, and this whole trial was thereafter classified as missing data. Furthermore, missing values occurred in four individuals in total 6 trials, due to reasons such as occlusion/missing of the markers during the side-bend movements and especially due to difficulties of synchronization during the single-leg balance trials. In the clinical study, no missing data occurred. All in all, missing data was not systematic and not more than 6 subjects per variable (81%).

### Psychometrics

The median and 5–95 percentiles of the Qinematic™ variables laboratory-based studies are presented in Table 2, together with the results from the Wilcoxon paired tests for testing if the data differed between occasions/measurement systems on group level (*p*-value), and all psychometric statistics: ICC_[3.1]_ with corresponding 95% confidence interval (95%CI), Spearman correlation coefficient (r), SEM and MCD.

**Table 2:** Results from the laboratory studies

	Data				Differences^1)^		Relative reliability	Relative validity		Absolute reliability	
	Reliability study		Validity study		Reliability	Validity					
	Qinematic™ TEST 1 Median (IQR)	Qinematic™ TEST 2 Median (IQR)	BTS-Elite Median (IQR)	Qinematic™ Median (IQR)	Qinematic™ T1 vs. T2 p-value^1^	Qinematic™ vs. BTS-Elite p-value^1^	Qinematic™ T1 vs. T2 ICC^2)^ (95%CI)	Qinematic™ vs. BTS-Elite r (p-value)^3)^	Qinematic™ vs BTS-Elite ICC^2)^ (95%CI)	Qinematic™ SEM^4)^	Qinematic™ MCD^5)^
Posture (Reliability study: n = 37; Validity study: *n* = 26)											
P-HL	1.34 (2.42)	2.14 (1.71)	−0.53 (2.45)	0.79 (2.85)	0.230	0.000	0.329 (0.01–0.59)	**0.487(0.014)**	0.418 (0.04–0.72)	1.388	3.846
P-HF	2.28 (6.52)	2.05 (5.46)	7.46 (5.89)	2.31 (5.63)	0.934	0.000	0.326 (0.01–0.59)	**0.723 (0.000)**	0.741 (0.49–0.64)	3.217	8.918
P-PP	0.45 (1.13)	0.49 (0.79)	−0.08 (1.52)	0.45 (0.90)	**0.028**	0.032	0.308 (−0.01–0.57)	0.270 (0.181)	0.342(0.04–0.64)	0.619	1.717
P-NA	−2.85 (6.26)	−2.76 (4.43)	–	–	0.054	–	0.723 (0.53–0.85)	–	–	1.876	5.200
P-HL	0.33 (0.32)	0.32 (0.24)	–	–	0.354	–	0.655 (0.42–0.81)	–	–	0.122	0.339
P-WB	48.52 (7.00)	46.95 (3.96)	49.16 (9.14)	48.96 (4.51)	**0.041**	0.259	0.231 (−0.10–0.52)	−0.073 (0.716)	−0.047 (−0.41–0.33)	3.372	9.349
Balance -Double stance (Reliability study: n = 37; Validity study: n = 26)											
DS-SA	0.10 (0.10)	0.10 (0.10)	0.12 (0.23)	0.10 (0.025)	0.591	0.069	0.125 (−0.20–0.43)	0.345 (0.084)	0.347 (−0.04–0.64)	0.632	1.752
DS-SVAP	0.70 (0.50)	0.60 (0.30)	3.36 (1.17)	0.55 (0.525)	0.532	**0.000**	0.013 (−0.20–0.43)	−0.273 (0.178)	−0.039 (−0.41–0.35)	1.094	3.033
DS-SVML	0.30 (0.15)	0.30 (0.30)	3.94 (0.88)	0.20 (0.1)	0.481	**0.000**	0.013 (−0.30–0.33)	0.159 (0.438)	0.038 (−0.35–0.41)	0.810	2.244
Balance - single stance, left (Reliability study: n = 37; Validity study: *n* = 24)											
SSL-SA	0.50 (0.70)	0.60 (0.40)	1.27 (1.85)	0.35 (0.58)	0.523	**0.000**	0.177 (−0.18–0.47)	0.301 (0.153)	0.111 (−0.30–0.49)	0.648	1.797
SSL-SVAP	1.20 (0.85)	1.30 (0.50)	8.89 (3.83)	1.25 (0.95)	0.521	**0.000**	0.228 (−0.10–0.51)	−0.115 (0.465)	−0.008 (−0.40–0.39)	0.503	1.393
SSL-SVML	1.40 (1.30)	1.30 (0.75)	8.89 (4.02)	1.25 (1.08)	0.631	**0.000**	0.162 (−0.17–0.46)	−0.087 (0.702)	−0.019 (−0.41–0.38)	0.684	1.896
Balance Single stance, right (Reliability study: n = 37; Validity study: *n* = 25)											
SSR-SA	0.50 (0.70)	0.50 (0.50)	0.87 (1.09)	0.40 (0.90)	0.432	**0.003**	−0.101 (−0.41–0.23)	0.260 (0.209)	0.422 (0.04–0.70)	0.907	2.515
SSR-SVAP	1.20 (0.75)	1.20 (0.55)	8.63 (3.99)	1.30 (0.90)	0.700	**0.000**	0.048 (−0.28–0.36)	0.316 (0.124)	0.011 (−0.38–0.40)	0.557	1.545
SSR-SVML	1.40 (1.00)	1.20 (0.75)	8.04 (4.41)	1.40 (1.30)	0.341	**0.000**	−0.094 (−0.40–0.23)	**0.640 (0.001)**	0.305 (−0.09–0.62)	1.016	2.816
Side-bending (Reliability study: n = 37; Validity study: *n* = 29)											
SB-LF	29.93 (3.09)	28.95 (3.21)	29.04 (5.78)	23.16 (5.83)	**0.000**	**0.000**	0.898 (0.81–0.95)	**0.943 (0.000)**	0.931 (0.86–0.95)	0.796	2.207
SB-RF	29.77 (3.33)	28.92 (3.82)	29.70 (5.13)	23.79 (6.38)	**0.018**	**0.259**	0.394 (0.08–0.63)	**0.888 (0.000)**	0.904 (0.81–0.95)	2.215	6.134
SB-%BW	85.01 (14.15)	84.18 (10.87)	–	–	0.105		0.693 (0.48–0.83)	–		5.700	15.799

^1)^ Wilcoxon signed rank test. ^2)^ ICC _[1, 3]_, ^3)^ Spearman correlation coefficient, ^4)^ SEM = 2*SD*ICC, ^5)^ MCD = √2 * SEM

* Numbers in bold: p < 0.05

Median and interquartile range (IQR) together with the reliability and construct validity statistics. Intraclass correlation (ICC _[1, 3]_) with significant ICC in bold, Spearman correlation (r) (p-value), Standard error of the measurement (SEM) and the Minimal Clinical Difference (MCD). Test-retest reliability statistics were calculated from the data from Test 1 (T1) and Test 2 (T2), and the construct validity statistics were calculated from data from Qinematic™ and BTS-Elite. See Table 1 for description of the variables

### Posture

When testing the test-retest reliability for the six variables related to posture, there were no significant differences between the occasions, except for two variables: pelvic medial displacement (P-PP), where the pelvic was positioned md 0.45 cm away from the midline on the first occasion and 0.49 cm on the second occasion (*p* = 0.028), and weight distribution where the md weight distribution was 48.52 at the first occasion and 46.95 the second (*p* = 0.041). Based on the ICC values, the test-retest reliability was classified as “fair” (ICC’s between 0.2 and 0.4) for the variables concerning posture (head medial, head forward, pelvic lateral and weight distribution), while for the variables concerning neck angle and height loss, the reliability was considered as “good” (ICC’s between 0.6 and 0.8). However, there were relative high SEM/MDC values for the variables related to posture, indicating that it is difficult to use these on an individual level (Table 2). The Bland-Altman plots did not show any sign of fixed or proportional bias.

In the construct validity study, there were significant differences between the two measurement systems for all variables except P-WB, weight balance during standing still. However, for this variable, the correlation between the two systems was below 0.2. The correlation between the two systems was rated “low” for variables P-HL and P-PP and “high” for the variable P-HF (Table 2).

In the clinical study, no differences between the LBP group and the control group were found for the variables related to posture (Table 3).

**Table 3:** POSTURE: Discriminative validity in patients with long-lasting low back (LBP), (*n* = 20), compared to healthy controls (*n* = 17). Median (IQR) and *p*-value of Mann Whitney U test. See Table 1 for description of the variables

	LBP	Controls	P-value
P-HL	5.13 (21.91)	−7.40 (24.84)	0.080
P-HF	63.36 (66.14)	37.79 (48.74)	0.517
P-PP	2.07 (10.02)	0.19 (14.27)	0.244
P-NA	0.57 (8.64)	−1.83 (5.88)	0.557
P-HL	5.00 (4.78)	4.12 (3.34)	0.177
P-WB	49.24 (4.40)	50.67 (5.06)	0.177

### Balance

When testing the test-retest reliability of the variables related to balance (sway area and sway velocity), there were no systematic differences between the two occasions, but the statistics showed ICC’s of <0.2 (Table 2) except for one variable (SSR-SA) in which the ICC reached 0.422 (95%CI 0.04–0.70). This, together with the large SEM/MCD, indicates poor reliability for the balance section in general.

In the construct validity study, the two measurement systems differed largely from each other for the variables related to sway area and sway velocity, with a higher sway area measured by the force plates. The correlation between these systems was also considered “poor”, since the ICC’s and Spearman coefficients only exceeded 0.4 in one out of six variables (Table 2). Comparing the two measurement systems, the force plates registered a 5–6 times higher maximal velocity in A/P and M/L directions and during the left leg stance task, and there were no correlations between the systems found. However, for sway M/L velocity during the single right leg standing task, there was a significant moderate correlation between Qinematic™ and BTS-Elite system (*r* = 0.640; *p* = 0.001). For all variables, the Bland-Altman plots showed clearly a systematic difference between these measurement systems, but the measurement error was not dependent of the measurement, i.e. there was no proportional bias.

In the clinical study, no differences between the LBP group and the control group were found for the variables in the balance section (Table 4), except for the sway A/P velocity during the right leg balance task (*p* = 0.031).

**Table 4:** BALANCE: Discriminative validity study in patients with long-lasting low back (LBP), (*n* = 20), and healthy controls (*n* = 17). Median (IQR) and p-value of Mann Whitney U test. See Table 1 for description of the variables

	LBP	Controls	P-value
Two leg balance			
D-SA	0.10 (0.10)	0.10 (0.15)	0.916
D-SVAP	0.70 (0.58)	0.50 (0.35)	0.326
D-SVML	0.30 (0.28)	0.20 (0.20)	0.341
Left leg balance			
L-SA	0.45 (0.88)	0.40 (0.60)	0.270
L-SVAP	1.4 (1.08)	1.0 (0.90)	0.257
L-SVML	1.55 (0.78)	1.40 (1.40)	0.940
Right leg balance			
R-SA	0.45 (1.33)	0.50 (0.65)	0.707
R-SVAP	1.55 (1.73)	0.90 (0.50)	0.013
R-SVML	1.95 (1.68)	1.80 (1.60)	0.707

### Side-bending

In the laboratory study, two of the three variables in the side-bending section showed significant differences between the occasions, indicating that the subjects increased their side-bending performance with around 1% of body height for both the left and right side (*p* < 0.019) at the second occasion. However, Table 2 showed that the relative test-retest reliability for side-bending to the left was “very good” (ICC = 0.898; 95%CI 0.81–0.95), while side-bending to the right was classified as “fair” (ICC = 0.394; 95%CI 0.08–0.63). The subject shifted their weight to the left side during the left side-bending in a similar way at both occasions: there were no systematic differences and the ICC was significant and classified as “good” (ICC = 0.693; 95%CI 0.48–0.83). The absolute reliability statistics (SEM and MCD) followed the same patterns for these three variables. The validity was for the two kinematic variables interpreted as “very good”, since both the Spearman correlation coefficients and the ICC were significant and very high (ICC > 0.88). There were, however, systematic differences found between the two systems: Wilcoxon rank sum test showed that the BTS-Elite system measured higher values than Qinematic™, indicating a systematic overestimation of Qinematic™ with around 6% of body height (Table 2). The Bland-Altman plots did not show any further proportional bias.

In the clinical study, the LBP group had a significant (*p* = 0.012) higher median (IQR) of side-bending to the left 29.4% of body height (IQR = 5.5) compared to the healthy controls which reached 26.7% of body height (IQR = 2.7). A higher value indicates a lower capacity. Similar results were found for the side-bending capacity to the right: the LBP patients reached 29.3% of body height (IQR = 5.0) and the controls 27.9% (IQR = 2.1), but here the difference was not significant (*p* = 0.244). There was no significant difference (*p* = 0.752) in the shift of body weight to the left between the groups; 82% (IQR = 14.8) in the LBP group compared to 85.6% (IQR = 16.0) in the healthy control group.

## Discussion

### Main findings

This paper explored the psychometric properties of a novel software program, Qinematic™, for measuring posture, balance and side-bending and showed poor/fair psychometric properties for the sections posture and balance, with large SEM/MCD, and poor discriminative validity. However, for the section “side-bending”, we found fair/very good reliability and very high validity.

### Results discussion

#### Reliability

The Kinect device has in previous studies been found to have satisfactory reliability [6, 7], which is why the poor reliability found in our study for balance and posture seems to relate to the individual variation and our results concur with other observational studies on intra-tester reliability [2–4], in which better results were found if video-analyses were used (i.e. excluding the individual variation). Higher reliability was found for side–bending, but only for the left side and it is difficult to know why only one side has acceptable reliability. The absolute measures of reliability were large for most of the variables, indicating that it is not possible to use these variables when monitoring a patient’s improvements over time.

#### Validity

In the laboratory study, we studied the construct validity, which is the type of validity that is applicable in situations in which there is no gold standard, and refers to whether the instrument provides the expected scores, based on existing knowledge about the construct (theory-based) [11]. We compared Qinematic™ data with the data obtained from the motion-capture system as done in previous studies [17, 18]. For the measures of postures, there were significant differences between the two measurement systems found for all variables except for the variable weight distribution, but for this variable the relative validity was very poor. Hence, the construct validity for variables measuring posture is low, and these results concur with a previous study in which high between device-differences (both fixed and proportional bias) were found in their Bland-Altman plots [18]. Despite this, they concluded, to our opinion incorrect, that the construct validity of the Kinect camera was excellent.

Concerning the balance variables, one force plate was used to measure postural sway as a “gold standard” for balance [19], but large sway does not always correspond to poor balance [20]. Hence, it could be difficult to measure balance capacity using sway only. The software program used in Qinematic™ calculates the position of the CoM, based on the body contours collected with the Kinect camera and uses the projection of this point to the ground to estimate the position of the CoP, while the force platform measures the CoP directly, and this measure contains also muscle forces applied to the floor. However, both systems aim to measure the construct “sway” and should give correlations above at least 0.6. This was not the case for the variables related to balance, and these results do not correspond to previous studies [6, 7, 18] which all reported higher correlations between CoM measured with a Kinect camera and a motion capture system. The reasons for this discrepancy between our and previous studies are several. In our study, we included subjects with ongoing pain, we used force plates for the calculation of CoP, we used a high sampling frequency, we used both absolute and relative statistics and we used standardized instructions, which are all factors that differ from most of the other studies. One important different is the short measurement time of the Qinematic™ system, which is much shorter compared to the Kinect systems used in previous studies. A systematic review of the test-retest reliability of CoP-variables concluded that the reliability of CoP varies depending on the design of the study and which variable of CoP to be evaluated. To achieve good reliability the sampling duration is proposed to be between 90 s to 120 s [21], and this is much longer compared to the software of Qinematic™, in which data is collected only for 3–5 s when balancing in one leg and 5–8 s during the standing task. In a previous study, good validity was obtained only for balance tests in which the subjects were testing their limits of stability and in balance tests performed with the eyes closed leading to a large sway area and sway velocity [18]. In Qinematic™, the tests are not challenging for balance to a large extent, leading to a small postural sway for all subjects, hence low between-subject variation. This result then in relative large within-subjects variation and low ICC’s; hence, poor validity.

For the measures of side-bending, an earlier study found acceptable correlations found between the Kinect camera and a motion capture system (Pearson correlations were over 0.89), but low ICC’s (less than 0.3) in both patients with early Parkinson and healthy controls [5]. These results are in concordance with ours as we found a nearly six-degree difference between the systems (29% of body height in BTS-Elite vs. 23% in Qinematic™) together with high correlations. They concluded that Kinect is not able to collect the spatial characteristics with the same precision as the timing characteristics, which seems to be a reasonable conclusion also for our results. Moreover, a recent study showed that KinectOne could not track small changes in trunk motion, which seems to be in accordance to our results [22].

We also tested the discriminative validity and we found that Qinematic™ was not able to detect differences between LBP and healthy controls in the posture and balance sections. On the one hand, large variation within the groups could have made it difficult to find significant differences (type 1 error). On the other hand, we found that the point estimates did not differ between the groups indicating no clinical difference between the groups. As previous studies have found differences in posture and balance between patients with chronic LBP and healthy controls [23, 24], our results showed that that Qinematic™ is not sensitive enough for measuring differences in posture and balance for patients with LBP.

#### Methodological considerations

Some of the strengths in this paper are its use of three different data collections with different study designs including both healthy subjects and patients, and different psychometric statistics and the fact that we obtained consistent results over the three studies. These results were both in concordance and contradicted previous studies that have investigated the Kinect camera [5, 17, 18, 22, 25]. We believe that the discrepancies between our study and these previous studies depend mostly on methodological differences. No other studies have previously used the Qinematic™ software program and tested these specific short static movements in which the subject was asked to stand still, and other studies used movements in which the subjects were moving through a wide range of motion or challenged in their balance, and this has a large effect on the ICC’s. Moreover, most of the previous studies have built their conclusions about validity and reliability only on correlation measures, and did not taken into account the occurrence of systematic differences, or discussed the absolute reliability measures such as SEM and MCD. These papers did not take into account the information obtained from Bland-Altman plots or follow the recommendations on how to design a reliability and validity study as suggested by the Cosmin groups [26], except for one recent study which provided both absolute and relative reliability statistics [22]. Another strength of this present study is that none of the authors had any financial relationship with the companies involved.

In the construct validity part of the laboratory study, it was possible for nearly all of the variables to be measured in the same way with the two systems systematically. However, due to the nature of the data in the one leg balance tasks, there was a need for a synchronization of the start and stop of the movements, and we used both marker data and a visual analysis for this process, which could have introduced errors into the measurement. However, it is unlikely that this led to a systematic over/underestimation of the balance capacity, which was confirmed by the Bland-Altman plots, thus we believe we can trust our data. The reflective markers on the skin could, theoretically, have altered the calculations of CoM and body positions (i.e. mid-head) of Qinematic™, since the contours could be changed, especially the marker on mid-head. It is, however, difficult to test to what extent this occurred.

Concerning the subjects, the sample was large (> 100 subjects included), and was a convenience sample consisting of both males and females with a range between 15 and 69 years of age, which has to be considered as representative of a working-age population. On the other hand, most of the subjects were physically more active than a normal population visiting a physiotherapy clinic, which could impair the generalizability of these results to a patient population. Some of the subjects had suffered from pain, while the majority were pain-free the last week, and this could also be seen as representative of a general population. However, when sensitivity analyses were performed in which we calculated the psychometric properties for these two groups separately (physical active and non-active subjects; pain and pain-free subjects), no differences were found, indicating that these results are robust for several populations.

In validity and reliability studies there are recommendations for the sample size to be at least 50, but larger samples are preferred [26, 27]. The reason why no more than 30 subjects for each sub-sample were required was due to lack of time, and a study population of 50 or more was not considered realistic. However, our sample sizes were 2–3 times larger compared to the studies discussed earlier [5–7, 22] in which the sample size was less than ten subjects, except for a recent study, in which 20 subjects were included. We believe that a larger sample size in this present study would not have affected the results, except for narrowing the IQR and lowering the *p*-values. The use of non-parametric statistics can be discussed. Still, using parametric tests did not alter our results to a large extent. Since we calculated both ICC and Spearman correlations in the validity study, we are able to make comparisons with previous studies, and we retrieved nearly similar results. We have tested Bland-Altman plots for all variables, but there were no additional fixed or proportional biases found in these plots and, due to lack of space, these were not included in the paper.

#### Future studies

Qinematic™ has one more part, the motor control part, in which the subject performs two different types of squats. In our next paper, we will present the results of the same psychometric analyses together with the face validity of these tests. Although the results from the presented studies are somewhat disappointing, we believe it is important to reveal the results from this section as well, since dynamic tests like squats are regularly used in all kinds of settings: sports, rehabilitation, physiotherapy, etc., and could be of higher interest compared to the static tests of balance and posture.

We believe that Qinematic™ has the potential of adding important information and as a pedagogic tool in the clinic, and it is exactly the type of software that has been lacking [5]. However, in the current version of Qinematic™, it seems that the individual variation of performing the tests together with the measurement error during the tests of balance, posture and side bending is too large for monitoring an individual over time or comparing different populations on a group level. Perhaps the introduction of cut-offs for good/poor balance, posture and side-bending capacity into dichotomous variables could reduce the large inter- and intra-individual variation and could give directly valuable information to the clinician. However, it is a challenge to define these cut-offs, since no such information is available in the literature, and there is a need for prospective studies on this purpose.

## Conclusions

This paper shows that a novel software program based on the Kinect camera for measuring balance, posture and side-bending has poor psychometric properties, indicating that the variables on balance and posture cannot be used for monitoring individual changes over time or in research. Future research on the dynamic tasks of Qinematic™ is warranted.

## Abbreviations

A/P: Anterio-posterior
COM: Centre of mass
COP: Centre of pressure
COSMIN: Consensus-based standards for the selections of health measurement instrument
DS: Double stance
DS-SA: Posture- Sway area
DS-SVAP: Posture - Maximal Sway Velocity in anterior-posterior (AP) direction
DS-SVL: Posture - Maximal Sway Velocity in medio-lateral (ML) direction
GRF: Ground reaction force
ICC: Intraclass correlation coefficient
IQR: Inter quartile range
LBP: Low back pain
M/L: Medio-lateral
MDC: Minimal detectable change
P-HF: Posture -Head-Frontal deviation
P-HL: Posture -Head-Lateral deviation
P-HS: Posture - Height loss
P-NA: Posture - Neck angle forward
P-PP: Posture - Pelvic position
P-WB: Posture - Weight distribution
REL: Reliability
SB-%BW: Weight distribution Left
SB-LF: Fingertips Left - distance to floor/body height
SB-RF: Fingertips Right - distance to floor/body height
SEM: Standard error of measurement
SSL: Single stance left
SSL-SA: Balance -Sway area
SSL-SVAP: Balance - Maximal Sway Velocity in anterior-posterior (AP) direction
SSL-SVML: Balance - Maximal Sway Velocity in medio-lateral (ML) direction
SSR: Single stance right
SSR-SA: Balance -Sway area
SSR-SVAP: Balance - Maximal Sway Velocity in anterior-posterior (A/P) direction
SSR-SVML: Balance - Maximal Sway Velocity in medio-lateral (M/L) direction
VAL: Validity

## Footnotes

1 ICC_[3.1]_ = (BMS – EMS) / (BMS + (k-1)EMS), in which BMS = Between targets mean square, EMS = Residual mean square, k = number of judges.

2
*SEM = 2*SD*ICC*

3 MCD = √2 * SEM
